# Risk factors and clinical features for post-transplant thoracic air-leak syndrome in adult patients receiving allogeneic haematopoietic stem cell transplantation

**DOI:** 10.1038/s41598-019-48308-9

**Published:** 2019-08-13

**Authors:** Yao-Chung Liu, Yi-Hsin Chou, Po-Shen Ko, Hao-Yuan Wang, Nai-Wen Fan, Chia-Jen Liu, Liang-Tsai Hsiao, Sheng-Hsuan Chien, Tzeon-Jye Chiou, Jin-Hwang Liu, Jyh-Pyng Gau

**Affiliations:** 1 Division of Haematology, Department of Medicine, Taipei Veterans General Hospital, Taipei, Taiwan; 2 Faculty of Medicine, National Yang-Ming University, Taipei, Taiwan; 3 Department of Ophthalmology, Taipei Veterans General Hospital, Taipei, Taiwan; 4 Division of Transfusion Medicine, Department of Medicine, Taipei Veterans General Hospital, Taipei, Taiwan; 5 Division of Haematology and Oncology, Department of Medicine, Taipei Tzu Chi Hospital, Buddhist Tzu Chi Medical Foundation, New Taipei City, Taiwan

**Keywords:** Cancer, Haematopoietic stem cells

## Abstract

Post-transplant thoracic air-leak syndrome (ALS) is rare but potentially life-threatening in patients receiving allogeneic haematopoietic stem cell transplantation (HSCT). Nevertheless, papers on thoracic ALS are limited, and this complication remains largely unknown. We reviewed 423 adult patients undergoing allogeneic HSCT from 2003 to 2014. Risk factors, clinical features and survival for thoracic ALS were collected and analysed. Thirteen out of 423 patients (3.1%) developed post-transplant thoracic ALS, including two ALS patients in the early phase. The median age at HSCT was 33 years among 13 patients with thoracic ALS. Male patients were predominant (69%). The median onset time was 253 days (range: 40–2680) after HSCT. Multivariate analysis revealed that grade III–IV acute graft-versus-host disease (GVHD) (*p* = 0.017), extensive chronic GVHD (cGVHD) (*p* = 0.019) and prior history of pulmonary invasive fungal infection (*p* = 0.007) were significant risk factors for thoracic ALS. In patients with cGVHD, those with thoracic ALS had a significantly worse survival than those without thoracic ALS (*p* = 0.04). Currently, published data analysing and exploring post-transplant thoracic ALS are limited. Our study employed a large patient cohort and determined the risk factors and clinical features for post-transplant thoracic ALS.

## Introduction

Patients receiving haematopoietic stem cell transplantation (SCT) have a 40–60% risk of pulmonary complications that result in transplant-related death in 10–40% of patients^1–3^. Although post-transplant thoracic air-leak syndrome (ALS) is rare, clinically, it is potentially life-threatening^1,4–6^. The definition of thoracic ALS is all forms of thoracic air leakage, including spontaneous pneumomediastinum or pneumopericardium, subcutaneous emphysema, interstitial emphysema and pneumothorax^7^. In contrast to common post-transplant organ dysfunction or damage caused by acute or chronic graft-versus-host disease (aGVHD or cGVHD)^8–10^, invasive fungal and bacterial infection^11–14^ or severe non-infectious complications^15^, the incidence of post-transplant thoracic ALS is relatively low with various values reported (0.83%–3.08%)^5,16–19^. However, post-transplant thoracic ALS had a fatal outcome according to some reported studies^16–19^. Young age, male, multiple SCT, cGVHD and late-onset non-infectious pulmonary complications (LONIPCs), including bronchiolitis obliterans (BO), bronchiolitis obliterans with organizing pneumonia (BOOP) and interstitial pneumonia (IP), were most commonly associated with thoracic ALS^16–18^. The clinical features, risk factors, and pathophysiology have rarely been explored for post-transplant thoracic ALS, especially in the late-onset group. However, the correlation between thoracic ALS and GVHD or other pre-transplant risk factors remains unclear. In the study, we aimed to review adult patients undergoing haematopoietic SCT with a focus on the complications in our hospital.

## Materials and Methods

### Study patient population

This was a retrospective study to review 423 adult patients undergoing allogeneic haematopoietic SCT between January 2003 and December 2014 in the Blood and Marrow Transplant Center at Taipei Veterans General Hospital in Taiwan. All patients received regular follow-up until October 2015. The cohort of study patients and follow-up time were the same as those reported in our previously published article^20^. We reviewed pre-transplant and transplant-related clinical data, including age, sex, pre-transplant biological characteristics, disease diagnosis, comorbidities, type of transplant, HLA matching, conditioning regimen, GVHD and other clinical complications. The study complied with the institutional ethical committee in agreement with the Helsinki Declaration of 1975, revised in 2008, and this study has been approved by the Institutional Review Board at Taipei Veterans General Hospital (no. 2014-11-002CC). All methods for the research were performed in accordance with relevant guidelines and regulations of Taipei Veterans General Hospital in Taiwan. The institutional ethical committee waived the informed consent form.

### Definition of thoracic ALS

Thoracic ALS was diagnosed by chest X-ray films and/or computed tomography scans after patients complained of cough, dyspnoea, chest pain or chest tightness. Pneumothorax was detected as extra-alveolar air in the left and/or right lung field. Pneumomediastinum or pneumopericardium was defined as extra-alveolar air in the mediastinal or pericardial space. Subcutaneous emphysema or interstitial emphysema was defined as the presence of extra-alveolar air in the subcutaneous tissue or pulmonary interstitial space, respectively^7^. Iatrogenic thoracic ALS caused by trans-bronchial lung biopsy, insertion of the central vein catheter, trauma or mechanical ventilation was excluded.

### Transplant details and conditioning regimens

HLA typing of low to intermediate resolution for 6 or 8 alleles (HLA–A, –B, –DR or –C) was used to screen donors for SCT. The donor source was obtained from matched sibling donor and alternative donors, including unrelated donor, haploidentical donor or umbilical cord blood. Myeloablative conditioning regimens included busulfan (4 mg/kg/day for 4 days) combined with cyclophosphamide (60 mg/kg/day for 2 days) or total body irradiation (TBI) of 12 Gy combined with cyclophosphamide (60 mg/kg/day for 2 days). Fludarabine-based conditioning regimens were administered to elderly patients or those with comorbidities. Regarding prophylactic use of antifungal agents during allogeneic haematopoietic SCT, fluconazole served as standard prophylaxis, but our physicians would consider echinocandin instead of fluconazole in patients with previous mould infection or those at high risk for aspergillosis. Prophylactic use of antifungal agents was stopped after the engraftment of absolute neutrophil count.

### GVHD prophylaxis and treatment

Standard protocol with cyclosporine (i.v. 3.0 mg/kg/day in 2 split doses with the dose adjusted to maintain trough plasma levels at 100–250 ug/L) and short-term low dose methotrexate (15 mg/m^2^ on day +1 and then 10 mg/m^2^ on days +3, +6 and +11 after SCT) were adopted for GVHD prophylaxis^20^. In addition, recipients of unrelated donor transplants also received rabbit anti-thymocyte globulin (2 mg/kg/day for 3 days). The severity of aGVHD was graded according to the system of Glucksberg and Thomas. The severity of the cGVHD was determined by the NIH scoring system, and cGVHD was classified as limited and extensive disease^21–23^. Patients with aGVHD >overall grade II, extensive cGVHD or alloimmune lung disease typically received high-dose methylprednisolone (1–2 mg/kg/day).

### CMV reactivation

CMV is tested using a quantitative PCR method. In our hospital, we do not use routine prophylaxis of CMV reactivation after SCT^20^. Instead, pre-emptive therapy with ganciclovir is administered when CMV reactivation is detected. In addition, double-strength trimethoprim-sulfamethoxazole is prescribed within 3 months after engraftment and in parallel with immunosuppressive therapy for GVHD.

### BOOP/BO evaluation and diagnosis

BOOP is diagnosed as organizing pneumonia related to secondary reaction in chronic bronchiolitis. Clinical presence of chest computed tomography (CT) scan revealed bilateral, patchy, and asymmetric areas consolidation with subpleural or peribronchovascular distribution or patchy and asymmetric ground-glass opacity^19^. BO was diagnosed by clinical histopathological findings and obstructive ventilatory defects on chest CT, including areas of decreased attenuation of lung parenchyma, expiratory air trapping and subsegmental or segmental bronchial dilatation and peribronchiolar opacities^24^.

### Study endpoints and statistical analysis

Pre-transplant data and post-transplant complications are presented as the total number (*n*) and proportion (%). Data are presented as medians and interquartile ranges (IQR) for skewed data. We used the Mann–Whitney U test or Fisher’s exact test to compare patients with and without thoracic ALS. Overall survival (OS) was analysed using the Kaplan–Meier product–limit method. A log–rank test was used to compare survival curves for statistical significance. Odds ratios (ORs) and the 95% confidence interval (CI) were calculated using logistic regression models. We used multivariate logistic regression models to calculate odds ratios while adjusting for possible independent confounding factors. All risk factors with *p* < 0.1 in the univariate model were further entered into the multivariate analysis. All statistical testing was performed using 2-tailed tests; *p* < 0.05 was considered statistically significant. All analyses were performed using SPSS statistical software, version 22 (SPSS, Chicago, IL).

## Results

### Patient characteristics

In total, 423 patients receiving allogeneic haematopoietic SCT between January 2003 and December 2014 were analysed. The median age at SCT was 42 years (IQR: 30–51) in all patients. The median follow-up after allogeneic haematopoietic SCT was 19.8 months (IQR: 4.87–53.87). In total, 13 patients (3.1%) experienced post-transplant thoracic ALS. The median age at SCT in patients with and without thoracic ALS was 33 years (IQR: 27–46) and 42 years (IQR: 30–52), respectively. Male patients were slightly more predominant in the two groups. No statistically significant difference in biological data was noted between the two groups. Moreover, the use of conditioning, disease type at diagnosis, smoking history and numbers of transplants also showed no significant difference. Patients with thoracic ALS had a trend of an increased proportion of pulmonary invasive fungal infection (IFI) history compared to those without thoracic ALS (*p* = 0.064). Patients with aGVHD or cGVHD were significantly more predominant in the thoracic ALS group, especially those with grade III–IV aGVHD (46%; *p* = 0.013) or extensive cGVHD (46%; *p* = 0.01). In addition, six out of thirteen patients (46%) with thoracic ALS had significantly more pulmonary cGVHD than those without ALS (5%) (*p* < 0.001). CMV reactivation showed no significant difference between the two groups. The clinical characteristics were summarized in Table 1.

**Table 1:** Baseline characteristics of adult patients receiving allogeneic SCT.

Characteristics	Allogeneic SCT recipient (*n* = 423)^b^		*p*-value^c^
	No ALS (*n* = 410)	ALS (*n* = 13)	
Age at SCT, years	42 [IQR: 30–52]	33 [IQR: 27–46]	0.232
Sex, male	226 (55)	9 (69)	0.401
White cell count, x10^9^/L (4.5–11^a^)	3.42 [2.118–4.815]	4.11 [2.31–7.21]	0.295
Haemoglobin, g/l (M: 140–180, F: 120–160^a^)	97 [84–114]	112 [86–128]	0.217
Platelet, x10^9^/L (150–350^a^)	115 [54–173.5]	108 [13.7–176.5]	0.300
Lactic dehydrogenase, U/L (131–250^a^)	195 [163–243]	194 [161–306]	0.757
Albumin, g/l (37–53^a^)	40 [37–42]	40 [37–42]	0.841
Calcium, mmol/L (2.1–2.65^a^)	2.25 [2.18–2.33]	2.23 [2.15–2.3]	0.262
Cholesterol, mmol/L (3.24–6.22^a^)	4.22 [3.5–4.95]	4.64 [3.91–5.28]	0.254
TG, mmol/L (0.23–2.26^a^)	1.38 [0.89–2.09]	1.42 [1.27–1.89]	0.433
BUN, mmol/L (2.5–7.14^a^)	4.64 [3.57–5.71]	4.28 [3.39–5.36]	0.495
UA, mmol/L (0.11–0.42^a^)	0.3 [0.22–0.37]	0.24 [0.17–0.41]	0.690
Creatinine, μmol/L (M: 61.88–132.6, F: 44.2–106.08^a^)	67.18 [55.69–79.56]	71.60 [58.34–86.63]	0.673
Total bilirubin, μmol/L (3.42–27.36^a^)	8.55 [5.99–10.94]	10.26 [7.7–11.63]	0.101
Alanine transaminase, U/L (0–40^a^)	25 [18–40]	27 [18–47]	0.774
Aspartate aminotransferase, U/L (5–45^a^)	21 [16–28]	21 [17–29]	0.773
Alkaline phosphatase, U/L (10–100^a^)	72 [57–91]	74 [61–115]	0.401
Gamma-glutamyltransferase, U/L (4–60^a^)	28 [17–47]	25 [13–81]	0.875
Sodium, mmol/L (135–147^a^)	140 [139–142]	141 [138–143]	0.461
Potassium, mmol/L (3.4–4.7^a^)	3.9 [3.6–4.1]	3.6 [3.6–4.1]	0.289
Glucose, mmol/L (3.61–6.38^a^)	5.16 [4.66–5.83]	4.94 [4.38–6.60]	0.699
PT, sec (8.0–12.0^a^)	10.8 [10.3–11.3]	10.8 [10.3–11.6]	0.816
aPTT, sec (23.9–35.5^a^)	28.0 [25.9–31.5]	27.1 [26.2–30]	0.589
C-reactive protein, mg/L (0–5^a^)	4.3 [1.6–21.8]	11.6 [2.9–70.7]	0.092
Myeloid malignancy (%)	197 (48)	7 (54)	0.781
Donor source, alternative donors (%)	221 (54)	8 (62)	0.779
Fludarabine based conditioning (%)	83 (20)	3 (23)	0.733
TBI-based conditioning (%)	177 (43)	7 (54)	0.572
Myeloablative conditioning (%)	267 (65)	10 (77)	0.556
Smoking history (%)	38 (9)	0 (0)	0.618
Pulmonary IFI history (%)	12 (3)	2 (15)	0.064
Repeat SCT (numbers ≧ 2) (%)	41 (10)	1 (8)	1.000
Acute GVHD, grade III–IV (%)	66 (16)	6 (46)	0.013
Chronic GVHD (%)	166 (40)	8 (62)	0.047
Extensive chronic GVHD (%)	63 (15)	6 (46)	0.010
Chronic pulmonary GVHD	22 (5)	6 (46)	<0.001
CMV reactivation (%)	215 (52)	7 (54)	1.000

SCT, stem cell transplantation; ALS, air-leak syndrome; TG, triacylglycerol; BUN, blood urea nitrogen; UA, uric acid; PT, prothrombin time; aPTT, activated partial thromboplastin time; TBI, total body irradiation; IFI, invasive fungal infection; GVHD, graft-versus-host disease; CMV, cytomegalovirus.

^a^Normal range.

^b^Values are reported as the median [IQR (interquartile-range)] or n (%).

^c^Determined using the Mann–Whitney U test for quantitative data and Fisher exact test for categorical data.

### Incidence and outcome of ALS

The overall cumulative incidence for post-transplant thoracic ALS was 3.1% and 2.6% (11/423) in late-onset thoracic ALS (onset time >100 days). In the early-onset group (onset time ≤100 days) (*n* = 2), thoracic ALS occurred at post-transplant days +40 and +100. One patient had left pneumothorax, and the other patient suffered from bilateral pneumothorax and pneumomediastinum. At thoracic ALS, two patients had grade III aGVHD with involvement of the skin, gut or liver. The two early-onset patients died of multiple organ failure (MOF) related to aGVHD and infection within 30 days. In the late-onset group, 6 out of 11 patients only developed pneumothorax, 1 patient only had pneumomediastinum, and the other 3 patients suffered from mixed types of thoracic ALS. Six patients had extensive cGVHD at ALS, and 5 out of 6 patients improved after adequate drainage, oxygen use or pleurodesis. Despite improvement by initial management, 4 out of 6 thoracic ALS patients with extensive cGVHD died of MOF, recurrence or progression of thoracic ALS. Three late-onset patients had limited cGVHD at thoracic ALS, and 2 out of 3 patients improved after drainage or oxygen use. All three thoracic ALS patients with limited cGVHD ultimately died of MOF or progression of thoracic ALS. In the last two late-onset patients without cGVHD at thoracic ALS, one suffered from severe pneumonia and failure to drain. He died of MOF and progression of thoracic ALS. The other patient had no BOOP or BO and survived after drainage. Median time for onset of thoracic ALS was 253 days (range: 40–2680) in all 13 patients and 474 days in the 11 late-onset patients (range: 142–2680). Three patients experienced relapse of thoracic ALS at intervals of 2, 5 and 11 days after remission of the first events. Detailed descriptions are summarized in Table 2.

**Table 2:** Clinical characteristics and outcomes of patients with post-transplant thoracic ALS.

ID	Age/Sex	Onset (days)	Dx.	TBI	Type of ALS	Repeated ALS (interval)	PFT before SCT	Lung condition at ALS	aGVHD	cGVHD	Initial therapy*/response	Clinical outcome	Death cause
1	50/F	1013	CML	No	PT (right)	Yes (5 days)	Normal	BOOP	I (skin)	Ext (lung)	Drainage/ improved	Died (48 days later)	ALS
2	42/F	163	NHL	Yes	PT (right)	No	—	—	III (liver, skin)	Limit (skin)	Observation/progression	Died (2 days later)	MOF + ALS
3	33/F	2680	NHL	Yes	PT (right)	No	Normal	BOOP	III (gut, skin)	Ext (gut, lung, skin)	Drainage/ improved	Died (3 days later)	MOF
4	31/M	587	ALL	Yes	SE + PT (right)	No	Normal	—	—	Limit (skin, lung)	Drainage/ improved	Died (144 days later)	MOF
5	27/M	229	AML	No	PM + SE + PT (right)	No	Normal	—	III (liver, skin)	Ext (gut, liver, skin)	Drainage/progression	Died (21 days later)	MOF + ALS
6	26/M	253	NHL	Yes	PM + SE + PT (bilateral)	No	Normal	—	—	Ext (skin, lung, heart)	Observation/Improved	Alive	—
7	23/M	474	ALL	Yes	PM	No	—	IP	—	Ext (lung)	Observation/improved	Alive	—
8	65/F	40	AML	No	PM + PT (bilateral)	No	Normal	—	III (gut, skin)	—	Drainage/ improved	Died (27 days later)	MOF
9	53/M	100	PMF	No	PT (left)	No	Normal	—	III (liver)	—	Drainage/improved	Died (26 days later)	MOF
10	42/M	142	ALL	Yes	PT (right)	No	Normal	—	I (skin)	Limit (lung)	Observation/improved	Died (7 days later)	MOF
11	38/M	166	AML	No	PM + SE + PT (right)	No	Mild restrictive	—	—	—	Drainage/progression	Died (28 days later)	MOF + ALS
12	32/M	2100	ALL	Yes	PT (bilateral)	Yes (2 days)	Normal	BO	III (gut, skin)	Ext (gut, liver, skin)	Drainage + pleurodesis/ improved	Died (1527 days later)	MOF
13	18/M	544	AML	No	PT (left)	YES (11 days)	Mild restrictive	—	—	—	Drainage/ improved	Alive	—

ID, identification; M, male; F, female; Dx, diagnosis; TBI, total body irradiation; SCT, stem cell transplantation; ALS, air-leak syndrome; PFT, pulmonary function test; BO, bronchiolitis obliterans; BOOP, bronchiolitis obliterans with organizing pneumonia; Ext, extensive type; Limit, limited type; PM, pneumomediastinum; MOF, multiple organ failure; PT, pneumothorax; SE, subcutaneous emphysema; IP, interstitial pneumonia; AML, acute myeloid leukaemia; ALL, acute lymphoblastic leukaemia; CML, chronic myeloid leukaemia; NHL, Non-Hodgkin lymphoma; PMF, primary myelofibrosis.

*All patients with ALS received oxygen.

**Figure 1:**
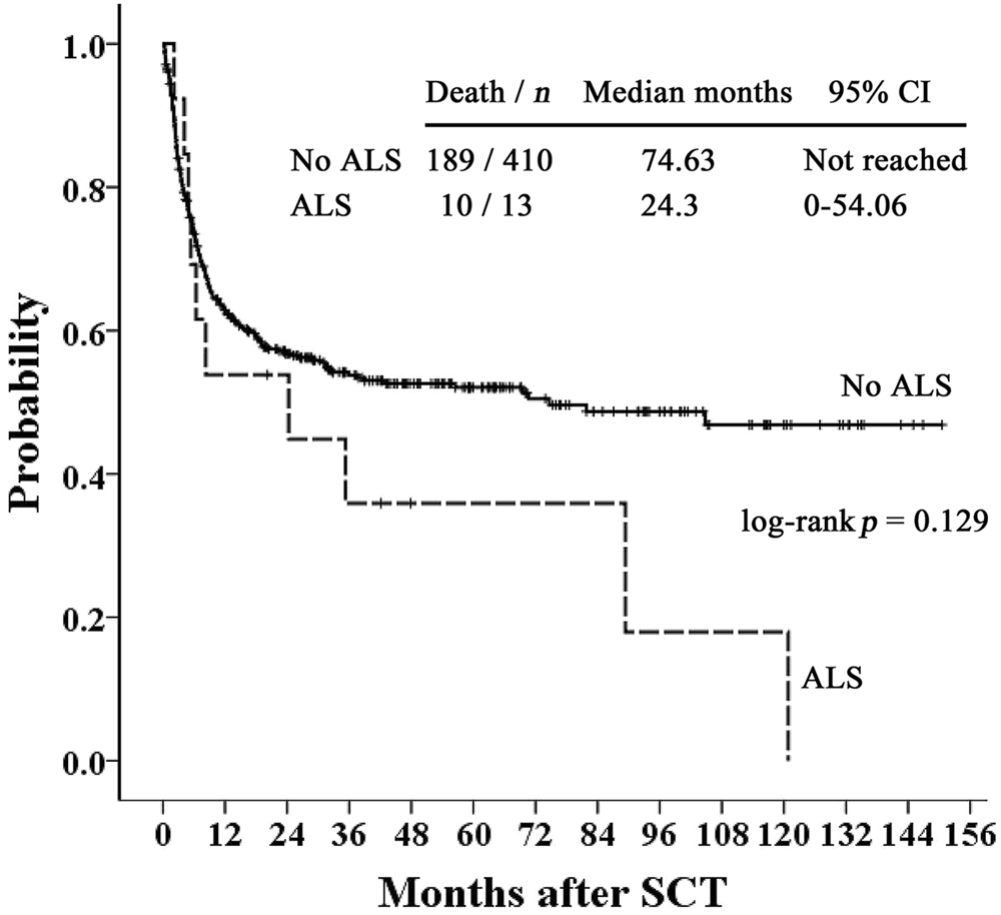
Kaplan–Meier curves of overall survival of patients with or without post-transplant thoracic ALS.

**Figure 2:**
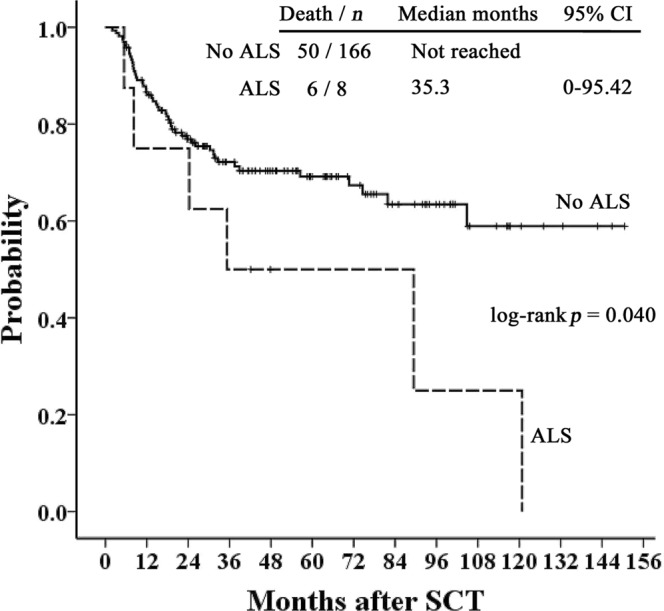
Kaplan–Meier curves of overall survival of cGVHD patients with or without post-transplant thoracic ALS.

**Figure 3:**
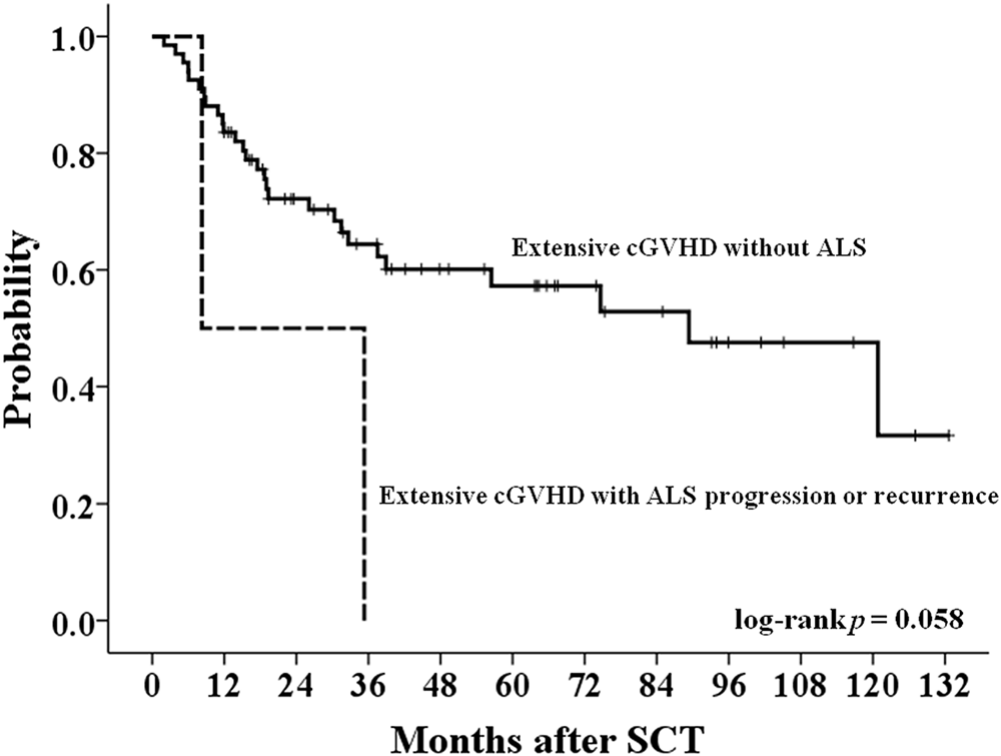
Kaplan–Meier curves of overall survival for extensive cGVHD patients without thoracic ALS or with thoracic ALS progression or recurrence.

For 350 patients who survived 3 months or more in our study, we also tested the association between thoracic ALS and LONIPCs by Fisher’s exact test. Among the 350 patients, 21 patients (6%) suffered from LONIPCs, including eleven BO, seven BOOP, and three non-classifiable IP patients. In 12 patients with thoracic ALS, four patients (33%) developed LONIPCs, which is more than those without ALS (17/321, 5.3%). A significant association was noted between thoracic ALS and LONIPCs (*p* = 0.003). In addition, there was no significant difference in post-transplant survival in patients with or without thoracic ALS (*p* = 0.129; Fig. 1). However, in 174 patients with cGVHD, survival was significantly poorer in patients with thoracic ALS than in those without thoracic ALS (*p* = 0.04; Fig. 2). For patients with extensive cGVHD with or without thoracic air leakage, thoracic ALS progression or recurrence contributed to a trend of poor survival based on Kaplan–Meier curves (*p* = 0.058; Fig. 3). However, for patients with extensive cGVHD with or without thoracic ALS, no significant difference was noted between these two groups (*p* = 0.783).

### Risk factors for post-transplant thoracic ALS

Of the 423 patients, 224 patients were younger than 43 years, and 10 of those patients (4.46%) developed thoracic ALS. In univariate analysis, age ≤42 years exhibited a trend as a risk factor (*p* = 0.094). After multivariate analysis, a significant trend (OR: 3.416; 95% CI: 0.853–13.676; *p* = 0.083) was revealed. However, pulmonary IFI history was an important significant predictor of thoracic ALS either in univariate (OR: 6.030; 95% CI: 1.203–30.240; *p* = 0.029) or multivariate analysis (OR: 11.845; 95% CI: 1.985–70.697; *p* = 0.007). We also evaluated associated post-transplant parameters, including aGVHD, cGVHD and CMV reactivation. In univariate analysis, grade III–IV aGVHD (OR: 4.468; 95% CI: 1.455–13.717; *p* = 0.009) and extensive cGVHD (OR: 4.721; 95% CI: 1.536–14.513; *p* = 0.007) were statistically significant. No statistical significance in CMV reactivation was found. However, the development of grade III–IV aGVHD (OR: 4.368; 95% CI: 1.301–14.669; *p* = 0.017) or extensive cGVHD (OR: 4.221; 95% CI: 1.261–14.126; *p* = 0.019) were important risk factors for thoracic ALS in multivariate analysis. The results are detailed in Table 3.

**Table 3:** Risk factors related to post-transplant thoracic air-leak syndrome.

Predictive variables	Univariate analysis		Multivariate analysis	
	OR (95% CI)	*p*-value	OR (95% CI)	*p-*value
Age ≦ 42 years	3.053 (0.828–11.255)	0.094	3.416 (0.853–13.676)	0.083
Sex (Male)	1.832 (0.555–6.044)	0.320		
Myeloid malignancy	1.261(0.417–3.818)	0.681		
Unrelated donor source	1.368 (0.440–4.253)	0.588		
Fludarabine based conditioning	1.182 (0.318–4.391)	0.803		
TBI-based conditioning	1.536 (0.507–4.650)	0.448		
Myeloablative conditioning	1.785 (0.484–6.591)	0.384		
Multiple SCT (numbers ≧ 2)	0.750 (0.095–5.916)	0.785		
Previous pulmonary IFI	6.030 (1.203–30.240)	0.029	11.845 (1.985–70.697)	0.007
Acute GVHD (grade III–IV)	4.468 (1.455–13.717)	0.009	4.368 (1.301–14.669)	0.017
Extensive chronic GVHD	4.721 (1.536–14.513)	0.007	4.221 (1.261–14.126)	0.019
CMV reactivation	1.058 (0.350–3.203)	0.920		

OR, odds ratio; CI, indicates confidence interval; TBI, total body irradiation; SCT, stem cell transplantation; IFI, invasive fungal infection; GVHD, graft-versus-host disease; CMV, Cytomegalovirus.

## Discussion

Post-transplant thoracic ALS has been rarely described in previously reported articles, and a complete understanding of the clinical features, risk factors, incidence and outcome is lacking. Before 2000, only some case reports explored this rare complication associated with LONIPCs and GVHD after allogeneic haematopoietic SCT^25,26^. After 2000, only some case series reported the incidence of post-transplant thoracic ALS in paediatric and adult patients. The reported incidence of this complication in the literature published between 2000 and 2014 was approximately 0.83% to 3.08%, which was similar to that in our study (3.1%) (Table 4). Male and myeloid malignancies were predominant in patients with thoracic ALS compared to the reported articles by Sakai R. *et al*. and Moon M. H. *et al*. (Table 4). The median onset time was 253 days post-transplant, which is also similar to previous reports of late-onset complications^5,16–19^. Early-onset post-transplant thoracic ALS is rarely diagnosed and typically occurs within 100 day after SCT. Previously, some articles reported few cases with early-onset thoracic ALS with poor outcome and unclear aetiology^18,27^. In such cases of early-onset thoracic ALS after SCT, the pulmonary injury caused by early-onset aGVHD, CMV viraemia, and TBI might easily induce pulmonary tissue injury^18,27^. In our study population, two patients suffered from an early-onset thoracic ALS, and all died of MOF within one month after initial improvement of this complication. Reviewing the medical records revealed no irradiation exposure in conditioning, while prior CMV reactivation and grade III aGVHD at thoracic ALS were found (Table 2). Our patients with thoracic ALS were predominantly diagnosed with late-onset complications (*n* = 11) more than 100 days after SCT. The result was similar to other studies^5,16–19^. In our study, the median age of 13 patients with thoracic ALS was 38 years, which is significantly younger than those without thoracic ALS but older than those in the reported cases series (range: 22–36.3 years) (Table 4). According to the report by Sakai R *et al*., age <38 years was confirmed as an independent risk factor^16^. In other reports, post-transplant thoracic ALS typically occurred in young recipients (Table 4). In our study, age ≤42 years was a trend as a risk factor for thoracic ALS. We agreed that post-transplant thoracic ALS can occur in young SCT recipients, but more patients should be included in further analysis.

**Table 4:** Reported adult cases series of post-transplant thoracic ALS after 2000.

Reference	Incidence	Median age (years) (M:F)	Diagnosis (*n*)	Median days from SCT to ALS	Type of ALS (*n*)	LONIPCs at ALS (*n*)	Prior aGVHD (*n*)	cGVHD at ALS (*n*)	Risk factor	Death (*n*)
*Franquet T*. *et al*.^5^	3.08% (9/292 aged ≧ 19 years) (one hospital)	28 (8:1)	AML (2) ALL (2) NHL (2) HL (1) MDS (1) CLL (1)	390	PT (3) Mixed (3) PM/SE (3)	BO (9)	—	Yes	Prior cGVHD (HR 14.1)	2
*Sakai R*. *et al*.^16^	1.19% (18/1515; aged ≧ 15 years) (nine hospitals)	29.5 (16:2)	AML (9) ALL (5) CML (2) ML (2)	575	PT (7) Mixed (6) PM/SE (6)	BO/BOOP (9) IP (5)	Gr. 0-I (6) Gr. II-IV (12)	No (1) Limit (2) Ext (15)	Age <38 years (OR 3.55) Male (OR 4.95) SCT ≧ 2 times (OR 7.91) FK prophylaxis (OR 3.30) cGVHD (OR 13.48)	11
*Toubai T*. *et al*.^17^	2.35% (5/213; aged ≧ 15 years) (one hospital)	22 (4:1)	AML (1) ALL (1) CML (1) AA (1) MDS (1)	225	PM/BP (5)	BO (5) IP (5)	Gr. 0-I (5)	Ext (5)	No statistical analysis data	5
*Vogel M*. *et al*.^19^	2.0% (6/300; aged ≧ 14 years) (one hospital)	32.5 (3:3)	CML (3) SAA (1) MDS (1) NHL (1)	No mention	PT (1) PM/IE (3) PM/PP (1) IE (1)	BO/BOOP (6)	—	Yes	No statistical analysis data	4
*Moon M*. *H*. *et al*.^18^	0.83% (18/2177; aged ≧ 18 years) (one hospital)	36.3 (8:10)	AML (6) ALL (4) CML (5) SAA (1) MDS (1) CLL (1)	425.9	PT (10) PM/SE (2) Mixed (4) PP/SE (1) PM (1)	BO/BOOP (2)	Yes (11)	Yes (7)	No statistical analysis data	16

ID, identification; M, male; F, female; SCT, stem cell transplantation; ALS, air-leak syndrome; aGVHD, acute graft-versus-host disease; cGVHD, chronic graft-versus-host disease; Gr., grade; BO, bronchiolitis obliterans; BOOP, bronchiolitis obliterans with organizing pneumonia; IP, interstitial pneumonia; HR, hazard ratio; OR, odds ratio; Ext, extensive type; Limit, limited type; PM, pneumomediastinum; MOF, multiple organ failure; PT, pneumothorax; BP, pneumothoraces; IE, interstitial emphysema; PP, pneumopericardium; SE, subcutaneous emphysema; Mixed, PT with ME/SE/IE; FK, tacrolimus; AML, acute myeloid leukemia; ALL, acute lymphoblastic leukemia; CML, chronic myeloid leukemia; CLL, chronic lymphocytic leukemia; MDS, myelodysplastic syndrome; SAA, severe aplastic anemia; AA, aplastic anemia; ML, malignant lymphoma; NHL, Non-Hodgkin lymphoma; HL, Hodgkin lymphoma.

There were two reported articles that analysed the associated risk factors of post-transplant thoracic ALS^5,16^. In addition to young age (<38 years), male, multiple transplants, GVHD prophylaxis with tacrolimus and prior cGVHD were also confirmed as independent risk factors (Table 4). Other possible associations with thoracic ALS, including BO, BOOP, and IP, were described in case series or reports (Table 4)^25,28^. In our series, SCT recipients with thoracic ALS prominently experienced grade III–IV aGVHD (46%), cGVHD (69%) and extensive cGVHD (46%) compared to those without thoracic ALS (Table 1). In multivariate analysis, we further confirmed grade III–IV aGVHD and extensive cGVHD as independent significant risk factors. It has also been reported that pulmonary cGVHD is a significant risk factor for LONIPCs^24,29,30^. In our study, chronic pulmonary GVHD had a significant association with thoracic ALS. We also found that three out of six thoracic ALS patients with pulmonary cGVHD (50%) developed BO, BOOP or IP. Although the definite pathobiological mechanism is not well known, host-reactive donor T cells play an important role in chronic inflammation of the lungs, airway fibrosis and subsequent LONIPCs progression^16,31^. In our study, 4 out of 12 late-onset thoracic ALS patients experienced LONIPCs, and all 4 patients with LONIPCs suffered from extensive cGVHD at thoracic ALS (Table 2). Among 350 patients who survived 3 months or more in our study, 21 patients (6%) suffered from LONIPCs, and this value is slightly less than those in the reported articles^29,32,33^. However, patients with thoracic ALS had a significant association with developing LONIPCs. Extensive cGVHD could lead to a strong association between the progression of LONPICs and thoracic ALS. Moreover, pulmonary IFI could cause pulmonary damage and thoracic ALS^34,35^. Studies describing the relationship between pulmonary IFI and thoracic ALS are lacking, especially in patients receiving allogeneic haematopoietic SCT. In our study population, patients with previous pulmonary IFI had a high risk for thoracic ALS.

According to the article by Sakai R *et al*., the survival of patients with post-transplant thoracic ALS or mixed type thoracic ALS was significantly impaired^16^. The possible pathomechanism of pneumothorax with pneumomediastinum or subcutaneous emphysema or interstitial emphysema (mixed type) is related to traumatic alveolar ruptures with air leakage along bronchovascular sheaths and extending into the mediastinum by the blunt pulmonary interstitial emphysema^19,36^. The mixed type of thoracic ALS reflects the extent of pulmonary or thoracic tissue damage. Sakai R *et al*. asserted that this reason might reflect the very poor survival of mixed type thoracic ALS patients^16^. In our study, the mixed type occurred in 5 patients, and 4 out of 5 patients died of MOF and/or thoracic ALS (Table 2). The median survival in patients with thoracic ALS was worse than in those without thoracic ALS but demonstrated no statistical significance by Kaplan–Meier curve analyses. However, in patients with cGVHD, those with thoracic ALS had significantly poorer survival. The result was compatible with reported cases^5,17–19^. In addition, thoracic ALS progression or recurrence in patients with extensive cGVHD may have a worse outcome (Fig. 3). From the point of view of post-transplant complications, we would like to emphasize the cross influence of thoracic ALS and GVHD control. Although some thoracic ALS could be controlled by drainage or observation, the complication still clinically affects the treatment of GVHD or increases LONIPCs. Even after the recovery of thoracic ALS, patients might die due to aggravation of GVHD, infections or other pulmonary complications. Especially for extensive cGVHD patients with thoracic ALS progression or recurrence, these two critical factors will be an important cause leading to a poor outcome. Therefore, we consider that thoracic ALS in patients after allogeneic haematopoietic SCT is associated with GVHD and indicates a poor outcome, especially in the setting of cGVHD or thoracic ALS progression or recurrence. Although lung transplantation for post-transplant pulmonary complications was a final life-saving choice and yields good outcomes^37^ no thoracic ALS patient received lung transplantation in our study.

Our study shared the inherent common limitations of a retrospective study, especially given that patients with asymptomatic thoracic ALS could not be detected. Moreover, the small number of patients with thoracic ALS, which is related to the low incidence of this complication, limited detailed analysis in our study. However, our study provides comprehensive biological data, clinical analysis and a literature review for this rare post-transplant complication.

In conclusion, despite its low incidence, we confirmed the risk factors for post-transplant thoracic ALS, including grade III–IV aGVHD, extensive cGVHD and patients with pulmonary IFI history. Patients with young age also had a trend of a risk for developing thoracic ALS. Patients with thoracic ALS had a worse outcome, especially in patients with cGVHD. Prospective studies are needed to determine the aetiology and optimal management of thoracic ALS after adult allogeneic haematopoietic SCT.
